# Editorial: New insights and perspectives of polyphenols in nutrition

**DOI:** 10.3389/fnut.2023.1145135

**Published:** 2023-02-21

**Authors:** Pasquale Crupi, Stefania De Santis, Simona Lucia Bavaro, Daniela Fracassetti

**Affiliations:** ^1^Research Center for Viticulture and Enology, Council for Agricultural Research and Economics (CREA), Rome, Italy; ^2^Department of Interdisciplinary Medicine, University of Bari Aldo Moro, Bari, Italy; ^3^Department of Pharmacy-Pharmaceutical Science, University of Bari Aldo Moro, Bari, Italy; ^4^Institute of Sciences of Food Production (ISPA)-National Research Council (CNR), Bari, Italy; ^5^Department of Food, Environmental and Nutritional Sciences (DeFENS), Università degli Studi di Milano, Milan, Italy

**Keywords:** polyphenols chemistry, bioavailability and bioaccessibility, methods of analysis, antioxidant, anti-inflammatory

An emerging trend in food science is the link between food and health. Consumer concerns center around what is in their food, and especially how it can promote wellbeing. Thus, nowadays, food is considered not only a source of energy but also an affordable way to prevent future diseases (1). Chronic diseases, such as cardiovascular diseases, obesity, and cancer, which are considered the leading causes of death and disability worldwide, are mainly related to an inappropriate lifestyle characterized by reduced physical activity and an unhealthy diet (2). An etiopathogenetic role for inflammation and oxidative stress was recognized for most chronic diseases. Therefore, the study of food polyphenols is pivotal because of their relevant and well-known antioxidant and anti-inflammatory activities (3). Although many studies have investigated the role and properties of polyphenols in human health (4), some challenges remain: (a) the link between chemical composition and biological activity in target tissues is still underestimated; (b) the absorption mechanism of phenolic compounds and their metabolism is still not well established; (c) their bioaccessibility and bioavailability based on food and food processing factors, interaction with other compounds, and host-related factors are still unknown since the promising beneficial effects of polyphenols initially depend on whether their concentration in an *in vitro* or *in vivo* assay becomes available at the site of action in the human body. Therefore, this Research Topic aims to collect research related to *in vitro* and/or *in vivo* studies on the antioxidant, anti-inflammatory, and antitumoral activities of polyphenols from plant and food matrices and, particularly, the relationship between their chemical composition and structure and their biological activity in target cells and tissues.

The paper by De Santis et al. described the anti-inflammatory abilities of extra virgin olive oil (EVOO) in obese children. The authors validated their previous *in vitro* data, in an *ex vivo* study on peripheral blood mononuclear cells (PBMCs) from obese children. Specifically, the study reported on the ability of EVOO extract to reduce the pro-inflammatory CD14+CD16+ monocytes, and the secreted CCL2 and CCL4 chemokines involved in the recruitment of inflammatory cells. Moreover, the molecular profile of EVOO treated-PBMCs from obese children was significantly modulated in terms of metabolic and inflammatory pathways. Importantly, a good correlation between the chemical profile of EVOO and its biological modulations in terms of anti-inflammatory activity was highlighted. Khoo et al. investigated the protective effects of the Chinese-origin red pitaya peel and pulp extracts in terms of antioxidative properties, lipid-reducing capacity, and cytotoxicity. Betanin, total betacyanins, total anthocyanins, and the DPPH index of both red pitaya pulp and peel extracts were analyzed. The authors demonstrated a higher content of total betacyanins and total anthocyanins in the pitaya peel extract than in the pulp extract, even if for the former a lower DPPH was reported. Biological studies were used to demonstrate the *in vitro* efficacy of the antioxidant and lipid-reducing effects, and the low cytotoxicity of the pitaya extracts. The paper by Mehmood et al. focused on the inhibitory mechanism of certain phenols toward the xanthine oxidase (XO) enzymes whose overactivity is directly linked to an excess of uric acid leading to hyperuricemia (HUA), gout, and many other diseases. The authors applied *in vitro*, atomic force microscopy, ^1^H NMR analysis, and different computational methods to assess the inhibitory potential of eight structurally diverse phenols. The results showed the high variability of the selected phenols in inhibiting XO depending on the phenol considered. The computational methods revealed that phenols can bind to the surrounding amino acids of XO at the molybdenum site. Rasouli et al. reviewed the current trends in the application of polyphenols (PPs) to alleviate aflatoxins (AFs) toxicity. The emergence of AFs has raised food safety issues as AFs can cause damage to the liver, kidney, and gastrointestinal organs, and the development of cancer for long-term exposure. The authors first reviewed factors influencing AFs occurrence, frequency, and world distribution, and the association between AFs and the pathogenesis of human chronic diseases. Then, they reported how PPs-rich extracts are promising AFs detoxifying products with heterogeneous biological activities. PPs can be used for the decontamination of AFs either in human/animal bodies or production/storage sites. The manuscript by Su et al. investigated the metabolic and molecular regulation mechanisms of chlorogenic acids (CGAs). The authors evaluated the composition and content of three forms of CGAs in mature fruits of 205 peach cultivars. The content of CGAs during the growth and development showed a trend of rising first and then decreasing in all peach cultivars studied. Moreover, the contents of quinic acid, shikimic acid, *p*-coumaroyl quinic acid, and caffeoyl shikimic acid all showed similar patterns to that of CGAs, indicating they could be involved in the accumulation of CGA during the growth. In addition, eight structural genes and 15 regulatory genes revealed that the expression levels of two Pp4CL, two PpCYP98A, and four PpHCT genes were positively correlated with the content of CGA, indicating that these may be key genes for CGA biosynthesis. Finally, Izuegbuna provided an overview of the possible use of polyphenols in the management of cancer blood diseases. The author especially described how polyphenols are involved in intracellular pathways by the phosphatidylinositol 3-kinase/protein kinase B way or in the PI3K/Akt/mTOR pathway, which is involved in every step of carcinogenesis as well as in the response to chemotherapy. In addition, the author detailed the effects of the polyphenols in the different steps of the cell cycle (autophagy, apoptosis, cell cycle arrest, and kynurenine pathway), displaying how they can be used as immunomodulatory agents in combination with some established therapies to attenuate the kynurenine pathway or enhance cellular immunity in hematological malignancies. Furthermore, clinical studies of the last 10–15 years herein reported, point out that polyphenols play an active role in clinical response and hematological malignancies management, suggesting their possible use in combination with chemotherapy or maintenance therapy.

In conclusion, the papers published in this Reaserch Topic clearly show that many aspects must still be clarified and understood in the fascinating world of polyphenols and their human health benefits.

## Author contributions

PC wrote the introduction and the conclusion. SD, SB, and DF wrote the central part with comments on the cited papers and references. All authors contributed to the article and approved the submitted version.

## References

[1] Ibáñez C Valdés A García-Cañas V Simó C Celebier M Rocamora-Reverte L. Global foodomics strategy to investigate the health benefits of dietary constituents. J Chromatogr A. (2012) 1248:139–53. 10.1016/j.chroma.2012.06.008 22727325

[2] De Santis S Liso M Verna G Curci F Milani G Faienza M F. Extra virgin olive oil extracts modulate the inflammatory ability of murine dendritic cells based on their polyphenols pattern: correlation between chemical composition and biological function. Antioxidants. (2021) 10:1016. 10.3390/antiox10071016 34202671 PMC8300824

[3] Milella RA Antonacci D Crupi P Incampo F Carrieri C Semeraro N. Skin extracts from 2 Italian table grapes (Italia and Palieri) inhibit tissue factor expression by human blood mononuclear cells. J Food Sci. (2012) 77:H154–9. 10.1111/j.1750-3841.2012.02818.x 22860586

[4] Fraga CG Croft KD Kennedy DO Tomás-Barberán FA. The effects of polyphenols and other bioactives on human health. Food Funct. (2019) 10:514–28. 10.1039/C8FO01997E 30746536

